# Preliminary Comparative Study of Oral7® Versus Salt-Soda Mouthwash on Oral Health Related Problems and Quality of Life among Head and Neck Cancer Patients Undergoing Radiotherapy

**DOI:** 10.21315/mjms2018.25.5.8

**Published:** 2018-10-30

**Authors:** Norsa’adah Bachok, Biswa Mohan Biswal, Noor Hayati Abdul Razak, Wan Mohd Nazri Wan Zainoon, Kasmawati Mokhtar, Roselinda Abdul Rahman, Mohd Faizal Abdullah, Siti Mimi Nadiya Mustafa, Nawi Noza

**Affiliations:** 1 Biostatistics & Research Methodology Unit, School of Medical Sciences, Health Campus, Universiti Sains Malaysia, 16150 Kubang Kerian, Kelantan, Malaysia; 2 KPJ Ipoh Specialist Hospital, 30350 Ipoh, Perak, Malaysia; 3 School of Dental Sciences, Health Campus, Universiti Sains Malaysia, 16150 Kubang Kerian, Kelantan, Malaysia; 4 Department of Nuclear Medicine, Radiotherapy & Oncology, School of Medical Sciences, Health Campus, Universiti Sains Malaysia, 16150 Kubang Kerian, Kelantan, Malaysia

**Keywords:** radiation effects, artificial saliva, mouthwashes, xerostomia

## Abstract

**Background:**

This quasi-clinical trial compared the effects of Oral7^®^ and salt-soda mouthwash on the development of dental caries, salivary gland function, radiation mucositis, xerostomia and EORTC QLQ H&N C35 scores in head and neck cancer patients who underwent radiotherapy.

**Methods:**

We included patients with histopathologically diagnosed head and neck cancers who had received radiation, with an Eastern Cooperative Oncology Group (ECOG) performance status 0–1 and age range of 15–60 years. Patients with prior radiotherapy and chemotherapy, edentulous status, total parotidectomy, sicca syndrome or on xerosis-induced medications were excluded. We assigned 15 patients each to the Oral7^®^ and salt-soda groups.

**Results:**

There was no significant difference in the mean Decayed, Missing and Filling Teeth (DMFT) score between groups. Head and neck cancer patients who were on Oral7^®^ had a significantly better quality of life than those on salt-soda in relation to the swallowing problems, social eating, mouth opening, xerostomia and illness scales. Patients who were on Oral7^®^ had a significantly lower xerostomia score than patients on salt-soda mouthwash. Patients on Oral7^®^ had a significantly lower mucositis score in week 5–7 compared to patients in the salt-soda group.

**Conclusion:**

Oral7^®^ showed advantages over salt-soda solution in relation to reducing xerostomia, easing radiation-induced mucositis, and improving quality of life, despite the non-significant difference in the dental caries assessment.

## Introduction

Head and neck cancers are cancers originating from the oral cavity, lips, nose, nasopharynx, paranasal sinuses, larynx, oropharynx, hypopharynx, thyroid and salivary glands. Oral and oropharyngeal cancer has been diagnosed in an estimated half a million cases worldwide and its prevalence is increasing, making it become the sixth most common cancer in the world (1). In Malaysia, nasopharyngeal cancer (NPC) is the fifth most common cancer and the third most common in males (2). The incidence and prevalence of head and neck cancers vary according to aetiological and geographical location. Developing countries have a higher incidence of nasopharyngeal cancer than developed countries (2).

Most cases of oral and oropharyngeal cancer are diagnosed at an advanced stage, especially in the less developed world, despite the simple and uncomplicated accessibility of the oral cavity for regular check-up (1, 3). The main factor contributing to the advanced stage at diagnosis is late presentation of the patient (3). With the lack of cancer awareness, patients are unable to recognise the initial symptoms, unaware of the severity of these symptoms and do not think to link their symptoms to the possibility of cancer (4).

Radiotherapy is the predominant modality of treatment in the multidisciplinary management of head and neck cancers. Radiotherapy exposes a substantial proportion of the head and neck tissues, including the salivary glands, to ionising radiation. Exposure of major salivary glands to ionising radiation results in reduction of salivary flow (5). This decrease in salivary flow leads to symptomatic xerostomia and consequent alteration of the oral microenvironment, and thus may affect people’s social life due to effects on swallowing, speech and sleep, and may indirectly affect quality of life. Prolonged xerostomia leads to dysphagia, sticky mouth, oral infection and resorption of teeth and caries (6, 7). In extreme circumstances, xerostomia causes permanent loss of teeth and poor quality of life (6, 7). Another common side effect of cancer treatment is mucositis, which leads to remarkable pain, poor nutrition and even systemic infection because it predisposes the affected individual to infection by fungi, viruses and bacteria (6). Management of xerostomia consists of avoidance of exposure of the parotid gland to radiation and artificial saliva supplementation (8). Fox divided salivary enhancement therapies into topical and systemic therapies (9). Topical therapies include flavoured gums or lozenges, artificial saliva, oral gels, flavoured mouthwashes, anhydrous crystalline maltose and acupuncture; systemic therapies include pilocarpine, cevimeline, interferon α, bromhexine, anethole trothone, traditional Asian mixtures, essential fatty acids, LongiVital, Yohimbine and Infliximab. Mucin-based artificial saliva and chewing gums have been reported to prevent dental problems due to post radiation complications or severe xerostomia due to Sjogren’s syndrome (10).

In this study, we compared the effects of Oral7^®^ and salt-soda mouthwash on the development of dental caries in head and neck cancer patients pre- and post-radiotherapy in a clinical trial. The secondary endpoints of the study were to compare salivary gland function, radiation mucositis, xerostomia score and EORTC QLQ H&N C35 score. We hypothesised that head and neck patients on Oral7^®^ would develop less dental caries and xerostomia, and show less deterioration of quality of life compared to the salt-soda mouthwash group.

## Materials and Methods

This was a quasi-clinical trial comparing the effects of Oral7^®^ versus salt-soda mouthwash on salivary gland function, mucositis, caries experience and quality of life amongst head and neck cancer patients who were receiving salivary gland suppressive radiation therapy. Patients were pooled from oncology clinics and screened for the trial.

Random allocation to treatment was attempted using the random numbers generated at www.randomization.com website. A written consent form was signed voluntarily for participation in this trial without obligation. Our research was approved by the Human Research Ethics Committee of Universiti Sains Malaysia, serial number USMKK/PPP/JEPeM [246.3.(7)]. Patients with histopathologically diagnosed head and neck cancers, Eastern Cooperative Oncology Group (ECOG) performance status 0–1, age range of 15–60 years, and who were appropriate for salivary gland suppressive radiation were included in the study. Patients with prior radiotherapy and chemotherapy, edentulous status, total parotidectomy, sicca syndrome, and patients on xerosis-induced medications were excluded. A priori sample size calculation required 35 patients to be randomised to Oral7^®^ and 35 patients to salt-soda mouthwash.

### Radiotherapy Technique

All patients with radiation to the head and neck region that included a biologically effective dose of more than 40 Gy to the whole parotid gland were considered for this study. Hence all patients treated using parallel opposed field techniques and the unilateral wedge portal field were considered. Chemotherapy may or may not be part of the treatment regimen. Patients whose parotid gland was shielded or received less than the stipulated radiation dose were excluded from the study. In general, the dose per fraction was limited to 180–200 cGy per day, 5 days a week with 2 days weekend break using a 6 Mv linear accelerator. The overall treatment time was limited to 8 weeks and treatment breaks were kept minimum.

### Administration of Oral7^®^ Mouthwash

Oral7^®^ mouthwash was provided by MDB Marketing Sdn Bhd Malaysia in a 200 mL bottle. The same amount of mouthwash (15 mL) was taken into the mouth, swished for 5 min and spit out four times a day, starting from 1 week before radiotherapy until 6 weeks after radiotherapy, including weekends and holidays.

Oral7^®^ mouthwash is formulated with the natural enzymes glucose oxidase, lactoperoxidase, lysozyme and lactoferrin which are similar to those in natural human saliva. Oral7^®^ aims to reinforce the functions of natural saliva, in terms of its antimicrobial and moisturising properties, by supplementing the insufficient salivary enzymes in patients with xerostomia. By boosting the protective functions of saliva, Oral7^®^ may assist in the management of mucositis in cancer patients. Protecting the function of the taste buds, aiding in swallowing and speech, as well as increasing comfort in the oral cavity are some of the ways Oral7^®^ helps to provide a better quality of life for these patients. As it does not contain alcohol, Oral7^®^ mouthwash does not burn or sting the sensitive tissues in the patients’ oral cavity.

### Administration of Salt-Soda Mouthwash

The salt-soda mouthwash solution was a mixture of one tablespoon each of sodium chloride and sodium bicarbonate in four cups of water. It was prepared in sterile conditions by the hospital’s pharmacist and packed and dispensed to the patient in a 200 mL bottle. Fifteen mililitres of the salt-soda solution was taken by mouth and swished for five minutes and then spit out four times a day starting from 1 week before radiotherapy until 6 weeks following radiotherapy, including weekends and holidays.

### Evaluation of Dental Caries

All patients were evaluated by one of the dental co-authors using standard orodental evaluation criteria. The Decayed, Missing and Filling Teeth (DMFT) score was determined at the start, end and 6 months after radiotherapy. The assessment was blinded with regard to treatment groups. DMFT is a technique for statistically managing the number of decayed, missing, or filled teeth in the mouth. We identified and added each component separately, then added all subgroup DMFTs for each participant. Analysis was based on the mean number of DMFT per person.

### Evaluation of Salivary Gland Functions

The baseline unstimulated and stimulated saliva was determined and recorded. We used an SG Saliva Testing Kit to determine salivary functions. The same test was repeated at the end of radiotherapy and 6 months after radiotherapy.

Prior to the saliva test, patients were instructed not to smoke, consume food or drink, brush their teeth or use a mouthwash for at least 1 h prior to the scheduled appointment time. The patients were then asked to chew on a piece of wax to stimulate salivary flow. After continuous chewing for 5 min, saliva was collected into the collection cup. The quantity of saliva was measured in mL after 5 min stimulation.

The subjective grade of xerostomia was documented using a validated xerostomia questionnaire developed by Eisbruch et al. (8) with permission to translate into the Malay language. The xerostomia questionnaire comprises eight items that are rated 0 to 10 regarding the difficulty of speaking, chewing, swallowing, dryness of mouth, fluid intake and sleeping.

### Evaluation of Mucositis

Radiation-induced mucositis was recorded by one of the co-authors using the Radiotherapy and Oncology Group (RTOG) mucositis score (11). Erythema is graded as grade 1, patchy mucositis as grade II, confluent mucositis as grade III and ulceration as grade IV. The measurement was repeated every week and at the end of radiotherapy.

### Evaluation of Quality-of-Life

The EORTC QLQ H&N 35 quality of life questionnaire was filled in by the patients in the Malay language. The questionnaire was developed by the European Organisation for Research and Treatment Cancer (12) and a Malay version is available. It consists of 35 items that are divided into 18 scales: pain, swallowing, sensory problems, speech problems, trouble with social eating, trouble with social contact, reduced sexuality, teeth, opening mouth, dry mouth, sticky saliva, coughing, feeling ill, pain killers, nutritional supplements, feeding tube, weight loss and weight gain. The ECOG performance status was found to be suitable for the inclusion criteria. The quality of life questionnaire was repeated at the end of radiotherapy and 6 months following radiotherapy.

### Follow-up Policy

The patients were seen by clinical oncologists every week at the oncology clinic for RTOG mucositis and body weight assessment. The dental caries evaluation was done before starting radiotherapy, at the end of radiotherapy and 6 months after radiotherapy. Follow-up assessments were performed 6 weeks following radiotherapy and every 2 months thereafter for 6 months. All assessments were carried out by the respective clinicians blinded with regard the treatment groups.

### Statistical Analyses

Data was analysed using the Statistical Package for Social Science version 22. The demographic data and treatment parameters were tabulated, presented and compared using the chi-square or Fisher exact or independent *t*-tests. The differences in mean scores between the Oral7^®^ and salt-soda groups over time in relation to DMFT index, quality of life, xerostomia score and mucositis scoring were analysed using repeated measure analysis of variance (ANOVA). The results are presented as *F* statistics and *P*-values. The level of significance value was less than 0.05.

## Results

We randomly assigned 30 head and neck cancer patients to Oral7^®^ and salt soda mouthwash groups. Table 1 shows the socio-demographic background of the participants. There was no significant difference in socio-demographic background between treatment groups. The mean age of the Oral7^®^ group was 47.13 (standard deviation (SD) 15.77) years and that of the salt-soda group was 46.33 (SD16.00). Most participants had primary NPC.

### Evaluation on Dental Caries Experience

Table 2 shows the mean DMFT score of the Oral7^®^ and salt-soda groups. There was no significant difference in the mean DMFT score between the Oral7^®^ and salt-soda groups among head and neck cancer patients [*F* stat (df) = 0.120 (1,10), *P*-value = 0.736].

### Evaluation of Salivary Gland Functions

**Figure 1:**
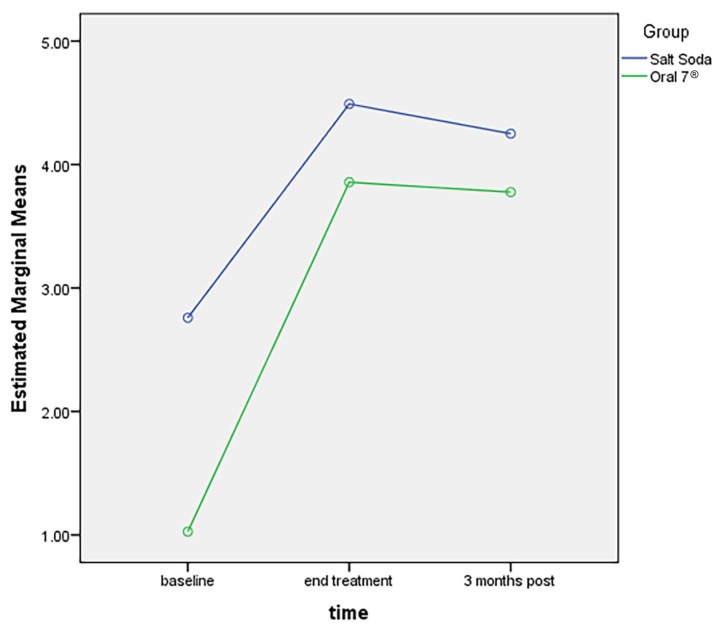
Comparison of xerostomia score between Oral7^®^ and salt-soda mouthwash groups among head and neck cancer patients who underwent radiotherapy

There was no significant difference in the mean saliva test score between the Oral7^®^ and salt-soda groups among head and neck cancer patients [*F* stat (df) = 3.080 (1, 26), *P*-value = 0.091]. Figure 1 shows the xerostomia score of the Oral7^®^ and salt-soda groups. Head and neck cancer patients who were on Oral7^®^ had a significantly lower xerostomia score than patients on salt-soda mouthwash [*F* stat (df) = 5.030 (1, 26), *P*-value = 0.034].

### Evaluation of Mucositis RTOG

**Figure 2:**
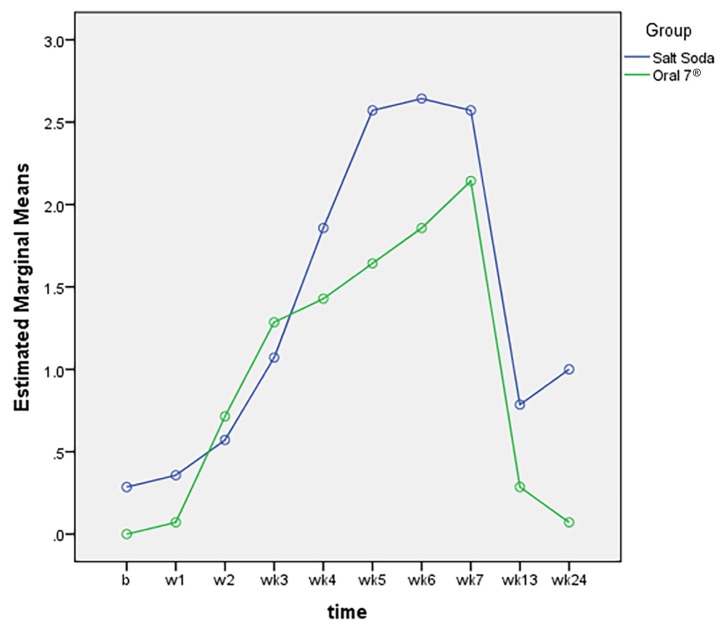
Comparison of radiation mucositis score between Oral7^®^ and salt-soda mouthwash groups among head and neck cancer patients who underwent radiotherapy

Figure 2 shows the radiation mucositis score of the Oral7^®^ and salt-soda mouthwash groups. Head and neck cancer patients who were on Oral7^®^ had a significantly lower mucositis score in weeks 5–7 compared to patients in the salt-soda group; *F* stat (df) = 195.240 (1, 26), *P*-value < 0.001.

### Quality of Life

There were significant differences in the quality of life EORTC QLQ H&N 35 scores between the Oral7^®^ and salt-soda groups with time in relation to swallowing problems [*P*-value = 0.024], social eating [*P*-value = 0.003], opening mouth [*P*-value = 0.001], xerostomia [*P*-value = 0.003] and illness [*P*-value = 0.006]. Head and neck cancer patients who were on Oral7^®^ had a significantly better quality of life than those on salt-soda according to the swallowing problems, social eating, mouth opening, xerostomia and illness scales.

There were no significant differences in pain [*P*-value = 0.236], sensory problems [*P*-value = 0.606], speech problems [*P*-value = 0.159], social contact [*P*-value = 0.193], sexuality [*P*-value = 0.061], dry mouth [*P*-value = 0.050], tooth problems [*P*-value = 0.918], coughs [*P*-value = 0.383], analgesic intake [*P*-value = 0.864], supplements [*P*-value = 0.415], weight loss [*P*-value = 0.990], weight gain [*P*-value = 0.141] and changes in BMI [*P*-value = 0.386] of quality of life scores between the Oral7^®^ and salt-soda groups.

## Discussion

Radiation for head and neck cancer may lead to multiple complications that will affect the patient’s quality of life. The common complications detected among patients are changes in taste sensation, dry mouth (xerostomia) and mucositis, which lead to a poor dental environment. Therefore, many treatments had been introduced to lessen the complications of radiation, either pharmacologically or non-pharmacologically. The present study tested a mucin-based artificial saliva versus salt-soda mouthwash. The results showed no significant difference in the dental status (DMFT score) between treatment groups. The lack of dental changes in this study could be due to the short follow-up. The earliest possible radiation-related caries may appear in the first 3 months following radiotherapy. A longer follow-up could show significant differences in dental caries evaluation. Furthermore, the findings indicate that there was the potential for a type II error of low statistical power, when in fact, a difference really existed. The reasons for the low power are the small sample size and small effect size, due to the large variation in the samples that do not have a substantial effect.

The saliva substitute can lessen xerostomia and had no significant side effect in the present study (13). Shiboski et. al reported that nine studies that tested the saliva substitute, found that it had mild effect on xerostomia and no effect on objective measurement of salivary hypofunction (10). However, most patients in the studies admitted that there was improvement of xerostomia compared to the baseline (10). Saliva substitutes are primarily used in xerostomia patient who are unable to stimulate saliva. They act as a moisturiser and lubricator to provide prolonged wetness of the oral tissues and to act as a protector against oral microorganisms (13).

Although this was a small study with a limited number of patients, the results show some of the expected benefits of artificial saliva (Oral7^®^). For example, Oral7^®^, which is mucin-based artificial saliva was better than salt-soda mouthwash at reducing the symptoms of xerostomia and mucositis and ultimately improving the patient’s quality of life. Our study showed the benefits of Oral7^®^ intervention in improving patients’ QOL in areas like swallowing capacity, social eating, mouth opening, xerostomia, and illness scales. The other differences between groups looked impressive but were not significant. Our results are consistent with most previous studies (10, 13–17). Most of these studies tested saliva substitutes that consisted of carboxylmethycellulose (CMC)-based, mucin-based, polyethylene-based and polyacrylic acid agents (10, 14). Eighty-nine percent of patients who received mucin-based artificial saliva experienced improvements in their xerostomia and 74% of them wanted to continue with supplement after the trial (13). These studies showed that mucin was widely accepted as an important component of saliva substitutes (15–17). More than 60% of the dry mouth patients preferred mucin chewing gum, as the mucin chewing gums were efficient at relieving the xerostomia at any time (15). Seventy-six percent of Sjogren patients who were experiencing dry mouth chose mucin lozenges rather than the placebo lozenges because the mucin lozenges improved oral dryness better during the day and night (16). A study showed that all tested items (gel, carmellose, oil and mucin) significantly improved the symptoms of xerostomia (17).

In terms of cost, both Oral7^®^ and salt-soda mouthwash are affordable. Amerongen and Veerman stated that the clinical use of saliva substitutes is financially possible as the costs of producing them are relatively low (14), while Dodd et al. stated that salt-soda mouthwash is cheap, easy to prepare and available anytime although there is no added benefit beyond typical systematic oral hygiene protocol (18).

The management of post-radiation head and neck cancer patient needs a multidisciplinary approach that involves preventive and palliative measures (10). The radiation oncologist, oral medicine specialists and dentists together provide supportive care for the patient during radiotherapy with the aim of preventing complications due to hyposalivation and radiation-induced osteonecrosis (10). Both preventive and palliative care are important so that the patients are able to pursue their life in the best way possible.

A limitation of this study was that fewer patients were recruited than originally intended. We encountered many technical difficulties during the recruitment period. We had great difficulty recruiting suitable participants who fulfilled our criteria and who were willing to participate. Most of our patients were receiving neo-adjuvant chemotherapy or palliative chemotherapy for advanced disease, and so did not meet our inclusion criteria. However, the results were promising and suggest that a larger scale and more robust study to look at the benefits of Oral7^®^ regarding mucositis and quality of life in head and neck cancer patients would be beneficial.

There are several confounding variables that were not controlled for in this study, such as radiation dosage and oral hygiene practices. We assumed that both groups of patients received similar oro-dental hygiene care that included mouth rinse, use of fluoride tooth paste and evaluation by our collaborating dentists.

In conclusion, it is evident that Oral7^®^ shows some advantages over traditional salt-soda mouthwash to ease radiation-induced mucositis and xerostomia and improve quality of life in head and neck cancer patients, despite the non-significant results in the dental assessment due to the small sample size and short follow-up. We suggest a further clinical trial with a larger number of patients from multiple centres and longer follow-up to verify the benefit of Oral7^®^. With an adequate sample size and follow-up of more than 6 months, such a study should be powerful enough to detect significant results.

## Figures and Tables

**Table 1:** Socio-demography characteristics and clinical background data of head and neck cancer patients who underwent radiotherapy (*n* = 30)

	Frequency (%)		*P*-value

	Oral7^®^ *n* = 15	Salt-soda *n* = 15	
Age (year)	47.13 (15.77)**	46.33 (16.00)**	0.891^
Sex			0.500#
Male	10 (66.7)	11 (73.3)	
Female	5 (33.3)	4 (26.7)	
Races			0.326#
Malay	11 (73.3)	13 (86.7)	
Non-Malay	4 (26.7)	2 (13.3)	
Diagnosis			0.705 *
NPC	9 (60.0)	10 (66.7)	
Others	6 (40.0)	5 (33.3)	
Stage			0.256*
III	8 (53.3)	11 (73.3)	
I/II/Unknown	7 (46.7)	4 (26.7)	
Treatment			0.500#
Concurrent Chemoradiotherapy	11 (73.3)	12 (80.0)	
Radiotherapy alone	4 (26.7)	3 (20.0)	

* Chi-square test

^ independent *t*-test

# Fisher exact test

** mean (standard deviation)

**Table 2:** Comparison of mean and 95% confidence interval of DMFT score between Oral7^®^ and salt-soda groups among head and neck cancer patients who underwent radiotherapy

	Mean (95% CI)		

	Baseline	Post-radiotherapy	3 months post-radiotherapy
Oral7^®^	13.7 (6.7, 20.7)	13.8 (6.7, 20.9)	13.7 (6.7, 20.6)
Salt-soda	13.7 (6.7, 20.7)	15.2 (8.1,	22.3) 16.8 (9.9, 23.8)

Repeated Measure ANOVA, *F* stat (df) = 0.120 (1, 10), *P*-value = 0.736
